# Developing and Evaluating Data Infrastructure and Implementation Tools to Support Cardiometabolic Disease Indicator Data Collection

**DOI:** 10.46292/sci23-00018S

**Published:** 2023-11-17

**Authors:** Mohammadreza Amiri, Suban Kangatharan, Louise Brisbois, Farnoosh Farahani, Natavan Khasiyeva, Meredith Burley, B. Catharine Craven

**Affiliations:** 1 KITE Research Institute, University Health Network, Toronto, ON, Canada; 2 ICON plc, Burlington, ON, Canada; 3 Spinal Cord Injury of Ontario, Toronto, ON, Canada; 4 Department of Medicine, Temerty Faculty of Medicine, Toronto, ON, Canada

**Keywords:** cardiometabolic risk, exercise, indicators, quality improvement, spinal cord injuries

## Abstract

**Background:**

Assessment of aerobic exercise (AE) and lipid profiles among individuals with spinal cord injury or disease (SCI/D) is critical for cardiometabolic disease (CMD) risk estimation.

**Objectives:**

To utilize an artificial intelligence (AI) tool for extracting indicator data and education tools to enable routine CMD indicator data collection in inpatient/outpatient settings, and to describe and evaluate the recall of AE levels and lipid profile assessment completion rates across care settings among adults with subacute and chronic SCI/D.

**Methods:**

A cross-sectional convenience sample of patients affiliated with University Health Network's SCI/D rehabilitation program and outpatients affiliated with SCI Ontario participated. The SCI-HIGH CMD intermediary outcome (IO) and final outcome (FO) indicator surveys were administered, using an AI tool to extract responses. Practice gaps were prospectively identified, and implementation tools were created to address gaps. Univariate and bivariate descriptive analyses were used.

**Results:**

The AI tool had <2% error rate for data extraction. Adults with SCI/D (*n* = 251; 124 IO, mean age 61; 127 FO, mean age 55; *p* = .004) completed the surveys. Fourteen percent of inpatients versus 48% of outpatients reported being taught AE. Fifteen percent of inpatients and 51% of outpatients recalled a lipid assessment (*p* < .01). Algorithms and education tools were developed to address identified knowledge gaps in patient AE and lipid assessments.

**Conclusion:**

Compelling CMD health service gaps warrant immediate attention to achieve AE and lipid assessment guideline adherence. AI indicator extraction paired with implementation tools may facilitate indicator deployment and modify CMD risk.

## Introduction

Cardiometabolic disease (CMD) refers to a clustering of interrelated risk factors that promote the development of atherosclerotic vascular disease and type 2 diabetes mellitus.^1^ These risks include abdominal obesity, insulin resistance, hypertension, low high-density lipoprotein cholesterol (HDL-c), elevated triglycerides, and physical inactivity.^1^ Elevated CMD risk leads to myocardial ischemia and infarction, angina, or stroke. Spinal cord injury or disease (SCI/D) causes complex changes in the affected individuals’ motor, sensory, and autonomic function, reducing their functional independence,^2^ which leads to an increased risk of CMD when compared to their age-matched peers without SCI/D.^3^ Although SCI/D may not uniformly increase CMD risk,^4^ physical inactivity and changes in lipid profile associated with dyslipidemia are key risk factors for CMD.^2^ The accurate identification of dyslipidemia and inactivity among individuals with SCI/D is critical to estimating CMD risk.^5^ Recently, two clinical practice guidelines for patients with SCI/D have been published to support the importance of CMD risk modification.^1^,^6^ The aforementioned guidelines paired with the high incidence of cardiovascular mortality necessitate action in the SCI rehabilitation community.^7^

Guideline adherence is often assessed through program audit and feedback, whereby clinicians collect key structure, process, and outcome indicators to assess performance at a single site or across a health system. Spinal Cord Injury Rehabilitation Care – High Performance Indicators (SCI-HIGH) project published CMD indicators in 2017.^2^ Routine collection of the SCI-HIGH intermediary outcome (IO) and final outcome (FO) indicators of CMD have not been previously implemented in Canadian tertiary SCI/D rehabilitation settings. Several factors may have influenced the low utilization of CMD indicators, such as patients and their caregivers prioritizing other areas of rehabilitation during their inpatient stay where patients were focused on (1) establishing bladder and bowel continence and (2) maximizing their neurologic and functional recovery and mobility prior to an anticipated recovery plateau at 12 to 18 months post injury.^8^ Assessments for CMD risk are often not implemented or deferred during the transitions from inpatient rehabilitation to community living setting. Delayed CMD risk assessments are missed opportunities to introduce primary and secondary prevention strategies and ameliorate future CMD-related morbidity and mortality. Recent publications highlight opportunities to improve preventive care from family physicians of individuals living with SCI/D^9^ by addressing knowledge gaps reported by primary care providers,^10^ and promoting primary and secondary prevention strategies. In general, lipid screening should begin earlier for patients living with SCI/D compared to their age-matched peers due to the link between dyslipidemia and duration of SCI/D. A description of a quality improvement (QI) process to facilitate CMD indicator implementation may provide valuable knowledge for rehabilitation facilities planning to implement CMD indicators in practice across care settings.

The enclosed QI project aimed to (1) describe the development of data infrastructure to enable CMD indicator data collection, (2) report the accuracy of data extraction using artificial intelligence (AI) software in a variety of clinical settings, (3) describe the development and refinement of implementation tools to support future provincial deployment of CMD indicators across settings among Ontarians with SCI/D, (4) explore current aerobic exercise (AE) adherence and recall of lipid profile assessments among adults living with SCI/D in inpatient tertiary rehabilitation or outpatient community settings, and (5) identify future steps to promote CMD risk reduction and tailor interventions for individuals with established intermediate CMD risk.

## Methods

This QI project was intended to support the future prospective longitudinal monitoring of adult Ontarians living with SCI/D and their indicators of CMD risk as they progress from inpatient rehabilitation settings to living in the community. A research ethics board waiver to proceed with this QI project was obtained (20-0007) from University Health Network (UHN). This project was conducted collaboratively between UHN and Spinal Cord Injury Ontario (SCI Ontario).

### Indicator surveys and AI platform for data extraction

A minimum data set including patient demographics, injury/disease onset, etiology of injury, and impairment level was created to support description of the individuals with SCI/D who participated in indicator data collection. CMD SCI-HIGH IO and FO indicator surveys^2^ that were previously designed for deployment in inpatient and outpatient settings were adapted by the QI project team to capture the demographic, impairment, and indicator data, and to record survey responses using optical mark recognition software. Digital data approvals for development and installation of Unstackr (formerly Reachlite^TM^) optimal mark recognition software on a unique secure server was obtained from UHN to ensure the vendor met established Ontario privacy and security standards. A total of 1000 mock surveys were used as training to fine-tune the Reachlite software algorithm for less than 5% error prior to clinical implementation.

### Data collection

Adults with SCI/D in inpatient rehabilitation completed the CMD IO indicator survey, whereas adults living with SCI/D in the community completed the FO indicator surveys (see eAppendices 1 and 2). The IO indicators evaluate the proportion of individuals with SCI/D who can articulate the AE component of the CMD health guidelines prior to rehabilitation discharge. The FO indicators describe the proportion of individuals with SCI/D with normal lipid profile at 18 months after rehabilitation admission and the proportion of individuals with SCI/D who are adhering to current AE guidelines at 18 months after rehabilitation admission. The IO or FO surveys were completed either in person or via telephone interview. Survey responses were recorded on paper or in digital format using an iPad Pro and Apple Pencil. At UHN, a kinesiologist obtained verbal consent and collected data using interviews and chart abstractions among inpatients and outpatients. For outpatients affiliated with SCI Ontario, a provincial intake coordinator collected data via phone interview. The provincial intake coordinator made a maximum of three attempts to contact potential participants by phone to obtain verbal consent for participation. The procedures for data collection and processes for responding to patient survey responses were standardized across participating organizations.

Following data collection, the digitized versions of completed surveys (i.e., scanned paper-based surveys or the portable document format [pdf] of iPad-based surveys) were imported into the AI tool (Reachlite) for data extraction. This AI tool allows the operator to extract CMD survey responses from an image of multiple-choice survey responses using optical mark recognition algorithms. As the responses are processed, Reachlite extracts data, recognizes the selected response, and associates the data with predefined data elements in the structured JSON format. The AI tool then maps the data from JSON format and pushes it into a comma-separated value output file (i.e., Excel file format) for local storage and statistical analysis.

### Flow diagrams and best practice implementation resources

We anticipated the need for routine processes for IO and FO survey administration, frequent detection of practice gaps during implementation, and a paucity of resources and implementation tools related to the new guidelines. We developed flow diagrams to support routine data collection and provided UHN and SCI Ontario staff with educational tools to share with participants and their care providers throughout QI project implementation. The implementation of best practices was supported by recently published guidelines.

The Paralyzed Veterans of America (PVA) CMD guidelines suggest individuals living with SCI/D should engage in at least 150 minutes of moderate intensity physical activity per week, broken into 30- to 60-minute bouts performed 3 to 5 days per week, or by exercising for at least 10 minutes, three time per day.^1^ However, Ginis et al.^6^ have argued that the 150 minutes per week guideline fails to consider the exercise capacity and limitations of individuals with SCI/D, specifically the risk of upper extremity overuse injuries, skin breakdown, autonomic dysreflexia, and overheating. Further, health benefits can be achieved with less than 150 minutes of exercise, suggesting a tailored approach to recommendations while considering feasibility and barriers to exercise is logical when addressing CMD risk.^6^,^11^ Given these considerations, Ginis et al. formulated the Canadian physical activity guidelines, which suggest 30 minutes of moderate to vigorous intensity AE three times per week.^6^ These guidelines (PVA CMD guideline and SCI Action AE component of the Physical Activity Guideline) were used to structure feedback and exercise counselling following IO and FO survey completion. The respondent's abilities, access to resources, and readiness to engage in exercise were considered when introducing AE recommendations.

During IO surveys, information regarding an inpatient's ability to recall if they were taught AE during their rehabilitation was recorded. The following questions were posed: “Has anyone in your healthcare team taught you about the need for long-term exercise (aerobic) for your heart health?” followed by “Has anyone in your healthcare team shown you how to perform exercise (aerobic) for your heart health?” (see eAppendix 1). These individuals were provided education using the exercise guidelines and CMD Toolkit Patient Handout (see eAppendix 3) if they were not aware of or did not know how to engage in AE.

**Figure 1.**
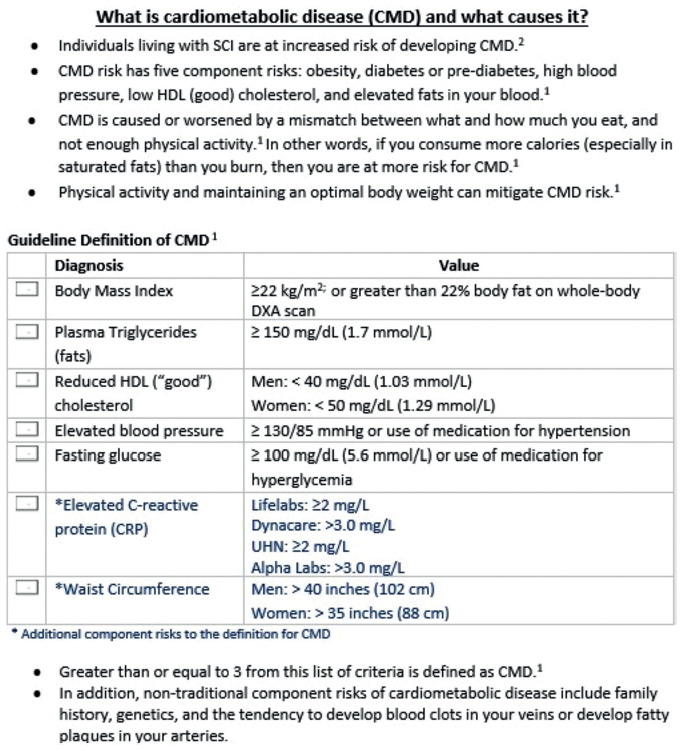
Introduction to cardiometabolic disease (CMD) risk factors and guideline definition.

**Figure 2.**
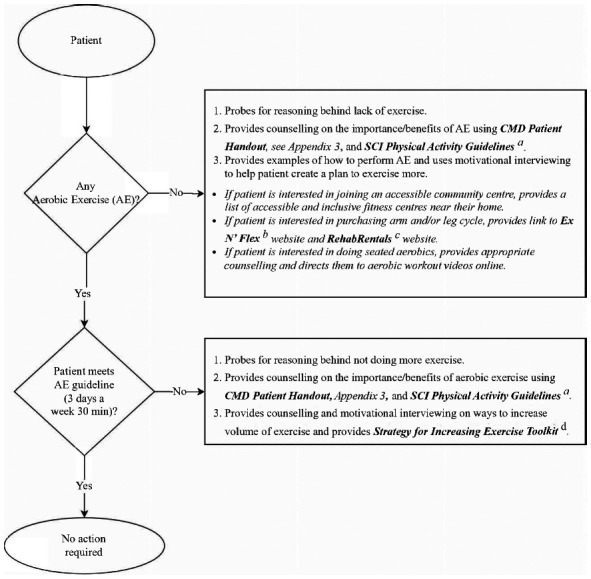
Algorithm for outpatient aerobic exercise guidelines and action plan. ^a^*SCI Physical Activity Guidelines: Physical Activity Guidelines for Adults with Spinal Cord Injury.* University of British Columbia Okanagan Campus. 2019. https://sciguidelines.ubc.ca/. ^b^Ex N’ Flex. https://www.exnflex.com/. ^c^Rehab Rental. http://www.rehabrental.ca/. ^d^*Strategy for Increasing Exercise.* Spinal Cord Injury Ontario. https://www.cortree.com/wp-content/uploads/courses/aging-and-heart-health/story_content/external_files/Strategy%20for%20Increasing%20Exercise.pdf. Note: Appendix 1 is provided as supplementary material (eAppendix 1; https://meridian.allenpress.com/tscir).

During the FO surveys, definitions were provided for AE and moderate to vigorous intensity exercise (see eAppendix 2). Participants were asked: “Do you do any moderate to vigorous-intensity exercise (aerobic) to maintain your heart health?” If respondents answered “yes,” they were then asked, “How many days per week, and minutes per day, did you engage in moderate to vigorous AE?” The patient's FO survey responses provided an opportunity to provide patients with educational resources such as the CMD Toolkit Patient Handout (see **Figure 1**, eAppendix 3) and SCI Physical Activity Guidelines,^12^ and provide feedback to motivate individuals to engage in AE and work toward AE guideline adherence (see **Figure 2**). The importance of self-monitoring and subjective ratings of exertion were emphasized to ensure safe and effective AE. This is particularly important given the propensity for patients with SCI/D to have low resting blood pressure; specifically, frequent postprandial hypotension was observed among older individuals, with higher lesions, and motor complete injury.^13^,^14^ Although exercise duration was documented in categories, absolute verbal answers allowed for tailored counselling. For example, if a respondent indicated they were performing 23 minutes of AE three times a week, they would be categorized in the “21-40 min/day” category. Although, this example respondent was meeting the AE volume guidelines, the counselling session's aim was to increase their exercise volume to 30 minutes per day. Following FO survey completion, participants were provided with the aforementioned resources (**Figure 1**, **Figure 2**, and eAppendix 3), notes about their CMD health, and recommended follow-up/action items (see eAppendix 4). Copies of the recommendations were placed in the individual's health record for review and reinforcement by their therapist, primary care provider, or physiatrist in the outpatient setting.

If during the FO survey, the respondent reported inactivity or AE below guideline levels, education was provided on the benefits of AE relative to CMD risk. The CMD Toolkit Patient Handout (eAppendix 3) describes CMD risk in lay language and highlights the importance of behaviour change. This tool was used to frame the conversation and was left with the patient as a resource to refer to post discussion. Inactive patients with chronic SCI/D participated in postsurvey motivational interviews using the Strategy for Increasing Exercise Toolkit^15^ to aid initiation of exercise or increasing AE levels based on their resources, impairments, and preferences. A list of accessible fitness centres was provided to those interested in group exercise programming or the interviewer would recommend local accessible community centres near the individual's home or online exercise options, wherever was most feasible for the participant.

There is evidence that upper-limb aerobic training is beneficial for improving cardiorespiratory fitness^16^ and increasing HDL-c levels, sometimes called “good” cholesterol, among individuals with SCI/D, leading to potential reductions in these CMD risk.^17^ Therefore, survey respondents were directed to arm cycling, wheeling, and seated aerobics as viable therapeutic options to explore. A majority of survey respondents did not have lower-limb function or access to lower-limb exercise machines such as a MOTOmed^®^. Survey respondents were asked to revisit the exercise equipment they own or were advised to purchase appropriate equipment.^1^ Two recommended vendors were Ex N’ Flex^18^ and Rehab Rental.^19^ However, participants were encouraged to compare prices and review similar equipment sold by other vendors. For individuals who reported enjoying being outdoors, the recommended exercise prescription was tailored to their preferred environments and appropriate dosing (i.e., rowing, swimming, or arm biking in the community).

### Lipid profiles

During the IO and FO surveys, participants were asked about their lipid profiles (see eAppendices 1 and 2). Inpatients were asked if they recalled having a lipid assessment done during their rehabilitation stay, whereas outpatients were asked about their recall of a lipid profile assessment within the last year. Due to several factors including varying lengths of inpatient rehabilitation stay, the length of time from inpatient rehabilitation discharge, and COVID impacts on outpatient clinic closures, participants were asked if their healthcare providers had told them about their lipid levels and/or recommended changes in their care. This allowed for a comparison between the patient's recall of a lipid assessment being collected compared to the actual results in their health record. However, to conduct this comparison, a classification of primary and secondary prevention was needed to interpret the results. Reference ranges and thresholds for abnormal values were identified, indicating the recommended management based on the actual collected bloodwork (not patient recall).

The Canadian Cardiovascular Society (CCS) uses the presence or absence of atherosclerotic cardiovascular disease (ASCVD) to determine primary or secondary prevention risk assessment for lipid screening. The CCS provides a list of at-risk individuals who should be screened for dyslipidemia in primary prevention settings.^20^ Our team modified and added the presence of these risk factors as “secondary prevention treatment targets” (see eAppendix 5). In the current project, primary prevention aims to prevent disease or injury before it occurs. This is done by altering unhealthy or unsafe behaviours that can lead to disease or injury and increasing resistance to disease or injury if exposure occurs.^21^ Secondary prevention aims to reduce the impact of a disease or injury that has already occurred.^21^ The PVA guidelines for HDL-c and triglyceride therapeutic targets 22 were used as main reference points, however a complete analysis was provided as a comprehensive report for healthcare providers. This report was compiled using values gathered from individual labs (Lifelabs, Dynacare, and UHN) as well as evidence-based data (see eAppendices 6 and 7). The survey administrators did not have access to, nor the expertise to calculate, estimates of 10-year CVD risk assessment, so the intermediate- or medium-risk values were chosen empirically for primary prevention reporting purposes where applicable.

**Figure 3.**
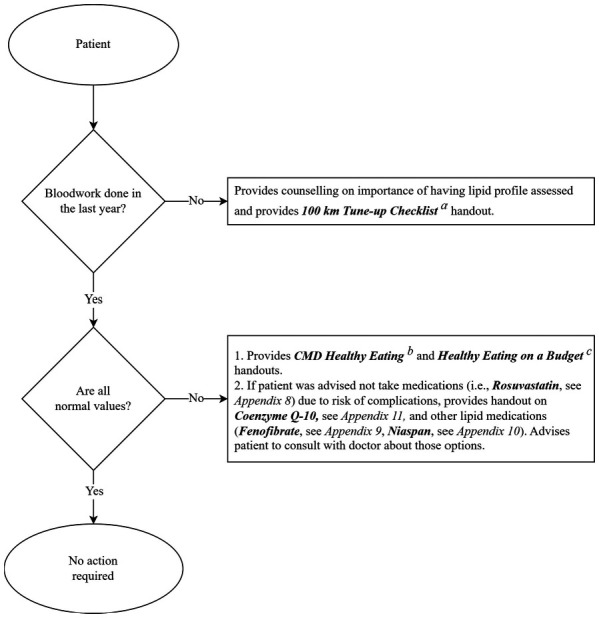
Algorithm for outpatient lipid profile assessment guidelines and action plan. ^a^100 Km Tune-up Checklist. Moore C. *100 Km Tune-up Checklist: Staying Healthy.* University Health Network. https://www.spinalcordessentials.ca. ^b^CMD Healthy Eating: Adapted from Cardiac College. https://www.healtheuniversity.ca/EN/CardiacCollege/Eating/. ^c^*Healthy Eating on a Budget.* Spinal Cord Injury Ontario. https://www.cortree.com/wp-content/uploads/courses/aging-and-heart-health/story_content/external_files/Healthy%20Eating%20on%20a%20Budget%20attachment.pdf. Note: Appendices 8-11 are provided as supplementary material (eAppendices 8-11; https://meridian.allenpress.com/tscir).

Counselling was provided to outpatients regarding their lipid assessments (see **Figure 3**). Survey respondents who reported they did not recall a lipid assessment or responded they did not know were counselled, and the 100 KM Tune-up Checklist was provided (**Figure 3**). The checklist highlights important items to keep track of on a routine basis in the context of healthy living with an SCI/D.^23^ Respondents who reported having had a lipid assessment and who reported abnormal results were provided handouts on healthy eating to educate them on approaches to reducing specific fats/lipids in their diet to improve their CMD risk profile. Participants were also introduced to Cardiac College as an important resource in cardiovascular health, particularly the sections on healthy eating. We anticipated that some primary care and physical medicine providers were unsure which medications to prescribe; thus, medication handouts were created to assist providers when discussing abnormal lipid profile results (see eAppendices 8-11). For example, Coenzyme Q-10 is prescribed to reduce the risk of myopathy for patients using statins. If a primary care provider had advised a participant to take a statin, the participant was also given a handout on this supplement to share with their primary care provider during the next visit.^24^

### Statistical analyses

Appropriate descriptive parametric and nonparametric statistics were used to describe survey respondents’ demographic and impairment characteristics. The status of AE and lipid profile assessments were stratified by inpatient (IO survey) and outpatient (FO survey) responses using both univariate and bivariate analyses. To examine whether there were differences in patient settings (i.e., inpatient vs. outpatient), we used a Pearson chi-square test of independence. For continuous variables, a Wilcoxon rank-sum test was used to identify the null hypothesis of “medians of the independent groups are the same.” All analyses were conducted using R Core Team (2021) version 4.2.2 (R Foundation for Statistical Computing, Vienna, Austria). An alpha level of ≤.05 was considered statistically significant for all statistical tests.

## Results

### AI for data extraction

The accuracy of the extracted data by the AI tool is presented in **Table 1**. In total, there were 5084 data fields in the IO surveys and 4953 data fields in the FO surveys requiring data extraction. The validation of the extracted data for the IO data had an error rate of 0.96%, whereas the FO surveys indicated an error rate of 0.86%. Additional details regarding the accuracy comparison between individual survey responses and individual data fields are presented in **Table 1**. The majority of collected data for both outcomes were collected digitally (IO: 66%; FO: 58%; *p* = .2).

**Table 1.** Evaluation of survey responses and individual data field accuracy using AI for data extraction

	Intermediary (*n* = 124)	Final (*n* = 127)
Individualized survey responses		
Number of forms, *n*	124	127
Number of questions, *n*	41	39
Total data fields, *n*	5084	4953
Total errors, *n*	49	92
Percentage errors, %	0.96%	1.86%
Individualized data fields		
Number of data fields per form, *n*	318	304
Total data fields, *n*	39,432	38,608
Total errors, *n*	49	92
Percentage errors, %	0.12%	0.24%

### Flow diagrams summarizing assessment and best practice integration

As this QI project aimed to provide concurrent implementation of best practices and indicators of quality care, flow diagrams were created to support staff who identified patient knowledge gaps during indicator survey completion. Two flow diagrams shown in **Figures 2** and **3** were developed to support clinical decision-making during survey data collection to ensure standard procedures are followed and the responses are customized to the patient's responses. The specific education interventions offered by the kinesiologist or intake coordinator, after survey completion, was contingent on the patient's specific indicator survey responses. The flow diagram in **Figure 2** highlights specific probing questions to be provided by the interviewer when speaking with the patient during the FO survey AE assessment. The flow diagram shown in **Figure 3** is used during the FO survey lipid profile assessment to provide specific recommendations based on the patient's responses to the probing questions.

### Survey participant characteristics

In total, 251 individuals with SCI/D (124 IO, mean age 61 years; 127 FO, mean age 55 years; *p* = .004 for age) completed the CMD surveys (see **Table 2**). The majority of survey participants were male (IO: 64%; FO: 67%; *p* = .5), with paraplegia (IO: 64%; FO: 55%; *p* = 0.16), with a preponderance of inpatients with nontraumatic injury (trauma prevalence, IO: 34%; FO: 67%; *p* < .01), with motor function below the neurologic level of injury (IO: 84%; FO: 62%; *p* < .01), and with touch sensation below the level of injury (IO: 89%; FO: 57%; *p* < .01). Although the mobility devices did not have similar proportions, manual wheelchairs were the most commonly used mobility device in the community (*p* = .05) and at home (*p* < .01). All of the IO data were collected at UHN, 79% of the FO data was collected at UHN, and 21% of the FO data was collected by SCI Ontario. The majority of data were collected digitally (IO: 66%; FO: 58%; *p* = .2).

**Table 2.** Demographic and impairment characteristics

Characteristic	*N* a	Intermediary (*n* = 124; 49%)	Final (*n* = 127; 51%)	Test statistic
Sex	243			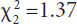 , *p*=.50^b^
Female		45 (36%)	41 (32%)	
Male		79 (64%)	85 (67%)	
Prefer not to say		0 (0%)	1 (0.8%)	
Not available		0	0	
Age, mean (*SD*)	244	60.7 (14.9)	55.4 (14.1)	t(249)=−2.87, *p*=.004^c^
Not available		0	0	
Length of cord injury, years	243			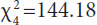 , *p*<.01^b^
<2		110 (95%)	23 (18%)	
2-5		2 (1.7%)	28 (22%)	
6-10		0 (0%)	11 (8.7%)	
11-15		1 (0.9%)	13 (10%)	
>15		3 (2.6%)	52 (41%)	
Not available		8	0	
Etiology	244			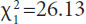 , *p*<.01^b^
Non-trauma		77 (66%)	42 (33%)	
Trauma		40 (34%)	85 (67%)	
Not available		7	0	
Impairment level	243			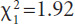 , *p*=.16^b^
Paraplegia		79 (64%)	70 (55%)	
Tetraplegia		45 (36%)	57 (45%)	
Not available		0	0	
Motor function: Yes	244	98 (84%)	79 (62%)	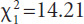 , *p*<.01^b^
Not available		7	0	
Touch sensation: Yes	243	103 (89%)	72 (57%)	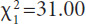 , *p*<.01^b^
Not available		8	0	
Mobility devices community	244			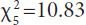 , *p*=.05^b^
Cane		1 (0.9%)	7 (5.5%)	
Manual Wheelchair		51 (44%)	48 (38%)	
Other		3 (2.6%)	9 (7.1%)	
Power Wheelchair		32 (27%)	43 (34%)	
Walker		22 (19%)	15 (12%)	
Walking without Assistance		8 (6.8%)		
Not available		7	0	
Mobility devices home	243			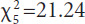 , *p*<.01^b^
Cane		4 (3.4%)	4 (3.1%)	
Manual Wheelchair		39 (34%)	55 (43%)	
Other		4 (3.4%)	5 (3.9%)	
Power Wheelchair		23 (20%)	38 (30%)	
Walker		37 (32%)	11 (8.7%)	
Walking without Assistance		9 (7.8%)		
Not available		8	0	
Site	251			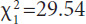 , *p*<.01^b^
SCI Ontario		0 (0%)	27 (21%)	
UHN		124 (100%)	100 (79%)	
Data collection method	251			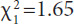 , *p*=.20^b^
Paper		42 (34%)	53 (42%)	
iPad		82 (66%)	74 (58%)	

*Note:* SCI Ontario = Spinal Cord Injury Ontario; UHN = University Health Network.

a *N* is the number of non-missing values.

b Pearson chi-square tests.

c Independent sample *t* test.

### Aerobic exercise

**Figure 4.**
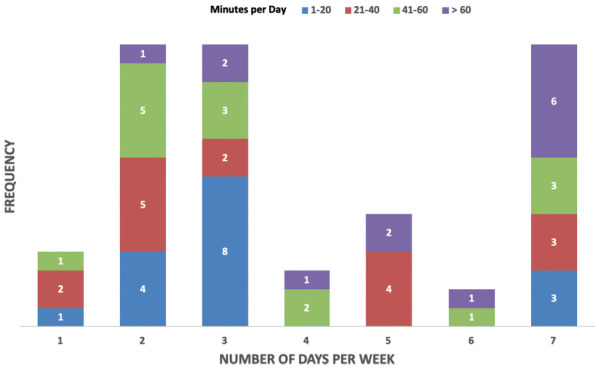
Frequency and duration of aerobic exercise among outpatients who reported at least one day per week of aerobic exercise.

It took a staff member approximately 30 minutes to administer each IO and FO survey and provide resources and training based on survey responses. Participants completing the FO survey were asked whether they “do any moderate to vigorous intensity exercise,” to which about 48% responded “yes” (see **Table 3**). Out of these participants, one in two were meeting the aerobic exercise elements of the physical activity guidelines (see **Figure 4**). This translated to 75% of outpatient FO participants being provided exercise guidelines and counselling related to CMD as per the processes outlined in **Figure 2**.

**Table 3.** Status of aerobic exercise among inpatients and outpatients

Characteristic	*N* a	Intermediary (*n* = 124; 49%)	Final (*n* = 127; 51%)	Test statistic
Any exercise	126			NA
Yes		NA	60 (48%)	
No		NA	65 (52%)	
Don't know		NA	1 (0.8%)	
Not available		NA	1	
Taught about need aerobic exercise	117			NA
Yes		16 (14%)	NA	
No		99 (85%)	NA	
Don't know		2 (1.7%)	NA	
Not available		7	127	
Shown how to perform exercise	16	11 (69%)	NA	NA
Not available		108	127	
Days per week	71			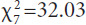 , *p*<.01^b^
1		0 (0%)	4 (6.7%)	
2		1 (9.1%)	15 (25%)	
3		0 (0%)	15 (25%)	
4		2 (18%)	3 (5.0%)	
5		0 (0%)	6 (10%)	
6		3 (27%)	2 (3.3%)	
7		2 (18%)	15 (25%)	
Don't know		3 (27%)	0 (0%)	
Not available		113	67	
Minutes per day	71			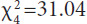 , *p*<.01^b^
<20		1 (9.1%)	16 (27%)	
21-40		3 (27%)	16 (27%)	
41-60		2 (18%)	15 (25%)	
>60		0 (0%)	13 (22%)	
Don't know		5 (45%)	0 (0%)	
Not available		113	67	
Type of exercise shown	10			NA
Arm cycling		4 (40%)	NA	
Cycling		3 (30%)	NA	
Other		1 (10%)	NA	
Walking		1 (10%)	NA	
Wheelchair pushing		1 (10%)	NA	
Not available		114	127	

*Note:* NA = not applicable.

a *N* is the number of non-missing values.

b Pearson chi-square tests.

The exercise frequency (*p* < .01) and duration (*p* < .01) of AE were dependent on the respondent's setting (inpatient or outpatient). Fourteen percent of IO respondents recalled being taught the need to perform AE for their CMD health as inpatients. Of this group, 69% of them recalled being shown how to perform AE (see **Table 3**). In total, 86% of inpatient participants were provided the physical activity guidelines and CMD Toolkit Patient Handout, as they did not recall being taught about AE or they were unsure what was taught.

### Lipid profile assessments

There was an interesting finding in both the IO and FO survey participants; there were discrepancies in what they reported and the frequency of actual tests conducted. Assessment of lipid profiles differed between the inpatient and outpatient setting (*p* < .01): only 15% of inpatients recalled the assessment, whereas 51% of outpatients recalled having their lipid profiles assessed since their discharge (see **Table 4**). Forty-three percent of IO respondents (inpatients) were not able to recall the information, whereas 16% of FO respondents (outpatients) did. The recollection of receiving lipid profile interpretation by a clinician also differed depending on the patient's setting (*p* < .01). Among inpatients, 39% reported not receiving a lipid interpretation, 30% could not recall the interpretation, and 30% reported receiving an interpretation. In contrast, 13% of outpatients reported not receiving any interpretation, 16% did not recall, and 70% reported receiving an interpretation of their lipid assessment. **Table 5** presents the proportion of individuals with abnormal values for specific lipid assessments for whom intervention was required to address their identified risk. The analyses indicate that abnormal values were prevalent and were not dependent on the setting (*p* > .1). For HDL-c, 86% and 50% of inpatients and outpatients (*p* < .05), respectively, had abnormal values. It is important to note that lipid data were not available for 72 outpatients due to restricted access to a shared results portal within their electronic health record.

**Table 4.** Status of lipid profile assessment

Characteristic	*N* a	Intermediary (*n* = 124; 49%)	Final (*n* = 127; 51%)	Test statistic
Received lipid profile assessment	241			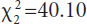 , *p*<.01^b^
Yes		17 (15%)	65 (51%)	
No		48 (42%)	42 (33%)	
Don't know		49 (43%)	20 (16%)	
Not available		10	0	
Received interpretations of lipid levels	108			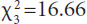 , *p*<.01^b^
Yes, abnormal		1 (3.0%)	13 (17%)	
Yes, normal		9 (27%)	40 (53%)	
No		13 (39%)	10 (13%)	
Don't know		10 (30%)	12 (16%)	
Not available		91	52	
Triglycerides test completed	179	38 (31%)	6 (11%)	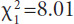 , *p*<.01^b^
Not available		0	72	
Cholesterol test completed	179	37 (30%)	6 (11%)	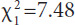 , *p*=.01^b^
Not available		0	72	
HDL-c test completed	179	37 (30%)	6 (11%)	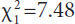 , *p*=.01^b^
Not available		0	72	
LDL-c test completed	179	38 (31%)	6 (11%)	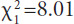 , *p*<.01^b^
Not available		0	72	
Non-HDL-c test completed	179	37 (30%)	6 (11%)	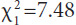 , *p*=.01^b^
Not available		0	72	
TC/HDL-c test completed	179	38 (31%)	6 (11%)	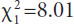 , *p*<.01^b^
Not available		0	72	

*Note:* HDL-c = high-density lipoprotein cholesterol; LDL-c = low-density lipoprotein cholesterol; TC = total cholesterol.

a *N* is the number of non-missing values.

b Pearson chi-square tests.

**Table 5.** Lipid profile abnormal values and necessity to take action

Characteristic	*N* a	Intermediary (*n* = 124; 49%)	Final (*n* = 127; 51%)	Test statistic
Triglycerides abnormal value	44	11 (29%)	2 (33%)	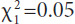 , *p*=.83^b^
Not available		86	121	
Triglycerides action required	44	8 (21%)	1 (17%)	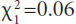 , *p*=.80^b^
Not available		86	121	
Cholesterol abnormal value	43	8 (22%)	1 (17%)	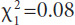 , *p*=.78^b^
Not available		87	121	
Cholesterol action required	43	8 (22%)	1 (17%)	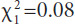 , *p*=.78^b^
Not available		87	121	
HDL-c abnormal value	43	32 (86%)	3 (50%)	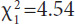 , *p*=.03^b^
Not available		87	121	
HDL-c action required	43	29 (78%)	3 (50%)	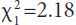 , *p*=.14^b^
Not available		87	121	
LDL-c abnormal value	44	17 (45%)	2 (33%)	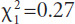 , *p*=.60^b^
Not available		86	121	
LDL-c action required	44	15 (39%)	2 (33%)	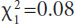 , *p*=.77^b^
Not available		86	121	
Non-HDL-c abnormal value	43	19 (51%)	5 (83%)	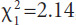 , *p*=.14^b^
Not available		87	121	
Non-HDL-c action required	43	16 (43%)	4 (67%)	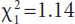 , *p*=.29^b^
Not available		87	121	
TC/HDL-c abnormal value	44	17 (45%)	4 (67%)	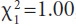 , *p*=.32^b^
Not available		86	121	
TC/HDL-c action required	45	14 (36%)	4 (67%)	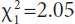 , *p*=.15^b^
Not available		85	121	

*Note:* HDL-c = high-density lipoprotein cholesterol; LDL-c = low-density lipoprotein cholesterol; TC = total cholesterol.

a *N* is the number of non-missing values.

b Pearson chi-square tests.

## Discussion

The products of this project are intended to enable future prospective longitudinal collection of CMD indicators in a variety of care settings and to track cohorts of patients prospectively transitioning through the health system. However, the data were obtained in a cross-sectional manner to inform near-term actions and process refinements. The accuracy of the AI tool supports the potential to scale the processes to support future national indicator implementation. The AI tool for data extraction was validated against the performance of trained personnel. The observed error rate was much lower than anticipated, confirming that OMR can accurately and rapidly extract electronic data into a comma-separated values format.^25^,^26^ Accurate and rapid extraction enabled the development of robust standard procedures and on-demand data analysis paired with best practice recommendations. Further, AI-powered tools can allow staff and administrators to better understand the opinions and preferences of their patients and make informed decisions based on the collated information.^27^

Physical inactivity is a common consequence of SCI/D; intrapersonal, interpersonal, institutional, community, and policy barriers often preclude routine participation in AE for individuals with SCI/D.^28^ Physical inactivity and other deleterious metabolic changes can lead to the development of obesity and other metabolic disorders, such as insulin resistance, dyslipidemia, and CMD.^29^ People living with SCI/D need a minimum of 30 minutes of AE a day, three times a week at a moderate-to-vigorous level to achieve a reduction in CMD risk factors.^12^ Regular AE is crucial for maintaining optimal body composition and preventing CMD disease onset in individuals with SCI/D.

The implementation tools created will address the AE and lipid profile enhancement needs of individuals living with SCI/D in real time. The hope is that the recommended behaviour changes and dietary interventions will modify CMD risk factors. The enclosed results, and observed high heart disease–related mortality, demonstrate an emerging CMD health crisis and the compelling need for routine AE prescription and counselling to ensure a larger proportion of the SCI/D population can adhere to established guidelines for CMD risk modification.

Many inpatients (85%) reported they were not educated about the value, frequency, or intensity of AE needed to maintain their heart health after discharge. Given the nature of the data collection, it is difficult to determine whether patients were not provided AE instruction, did not recall AE education provision, or whether AE recommendations were confused or combined in the patient's mind with exercise recommendations for functional or neurological recovery. The provision of educational materials, one-on-one instruction, and take-home recommendations following survey completion were intended to facilitate patient recall.

During inpatient rehabilitation, patients often experience an adjustment disorder related to the new onset of their disability, grief in the loss of their independence, and struggle to learn a myriad of new tasks. Polypharmacy post discharge^30^ and the rising mean age of nontraumatic injury^31^ may also contribute to some of the discrepancies between patient recall of AE instruction or lipid assessment and the greater frequency of lipid assessments conducted. Although many outpatients reported participating in AE, most were not adhering to the frequency, intensity, and duration of AE articulated in the physical activity guidelines. Common feasibility constraints were related to preference for exercise, location, and format as probed and revealed from the survey data collected. However, many additional systematic and policy barriers remain.^32^,^33^

We acknowledge that the approach and clinical strategies described here are intended to support routine AE and lipid assessments as a means of modifying CMD risk. These indicators are intended to be part of larger prospective programs of research to address CMD risk, recognizing that AE and lipid assessments are vital for early intervention from a population health perspective. Future deployment of the CMD indicators throughout participating sites of the SCI – Implementation and Evaluation Quality Care Consortium (www.sciconsortium.ca) is being planned with increased refinement of the supplementary materials to support broader reach.

As part of the motivational interviewing initiative, it was important for individuals to set realistic goals based on their current activity levels. For those who engaged in little to no AE, the guideline recommendation of 20 minutes of moderate-to-vigorous exercise two times a week was the initial focus to gain cardiorespiratory fitness,^12^ although the ultimate goal of achieving cardiometabolic health benefits through higher volumes of activity was emphasized throughout the interviews. We anticipated that most individuals would not be able to perform the recommended levels of exercise all in one bout, therefore we suggested that patients break their routine into multiple 10-minute bouts as a compensatory strategy for this barrier.

Clinical assessments, such as blood tests and diagnostic procedures, can be used to obtain objective data on markers of CMD risk.^29^,^34^ Diagnostic tools to assess levels of atherosclerosis such as carotid intima media thickness, coronary artery calcium scoring, and computed tomography angiography are all useful for measuring and tracking CMD progression.^35^ Exploring these new technologies congruently with previously known CMD risk factors may be crucial in preventing late diagnoses of CMD risk factors or an increase in fatal cardiac events.^35^

Lipid profile screening is an important aspect of managing CMD risk, although nutrition education alone may not be sufficient to improve or alter a lipid profile.^36^ Combining nutrition lifestyle changes with AE may be a more effective approach at reducing CMD risk.^12^,^37^ Changes in lipid levels, such as elevated levels of low-density lipoprotein cholesterol (LDL-c) and decreased levels of HDL-c, are associated with an increased risk of CMD disease. In a meta-analysis of 50 studies,^38^ individuals with SCI/D were noted to have significantly higher average total cholesterol to HDL-c (TC/HDL-c) ratios and significantly lower HDL-c levels, which illustrates the higher baseline risk of developing CMD. Regular lipid profile screening can help identify individuals with abnormal lipid levels and allow for early interventions to prevent the development of CMD risk. Patients with SCI/D need to understand their metabolic risk factors in order to take appropriate steps to manage their health. Further, there is evidence that individuals with SCI/D whose time since injury is less than a year and who have a motor complete SCI/D may have a higher risk of dyslipidemia.^39^ In this case, rehabilitation service providers and primary care providers need to be engaged in best practice implementation. To address this knowledge gap, it is important for healthcare providers to educate individuals with SCI/D about their lipid profile assessment and the potential risks associated with abnormal lipid levels. This can be done through patient education programs, informational materials, and regular discussions with healthcare providers.

Although not addressed in the current report, strength training is also very important for people living with a SCI/D aiming to improve muscle strength and aerobic capacity. Current guidelines recommend a target volume of three sets of ten repetitions for each major functioning muscle group for two days a week.^2^,^6^,^12^ Counselling on the benefits of strength training is important to overall physical activity interventions. Resources from SCI Action Canada (Home Strength-Training Guide for Paraplegia or Tetraplegia) can be utilized as these have most of the pertinent information needed for this population to engage in a strength-training program.^40^,^41^ In addition, UHN's Cardiac College has included information on purchasing resistance bands and extremity straps, which can be used to facilitate these intervention strategies.^42^,^43^

## Conclusion

The tested AI tool demonstrates excellent speed and accuracy of data extraction from the IO and FO indicator surveys. There is a significant opportunity to advance participation among inpatients with SCI/D in AE training using the developed implementation tools to reduce CMD risk. Inclusion of AE and lipid management instructions in patient-oriented discharge summaries may enhance patient recall and adherence. AE guideline adherence and routine lipid assessments are integral to CMD risk modification, and provision of education resources and recall aids may help to modify CMD risk after SCI/D. By increasing knowledge about lipid profile assessment and the risk reduction associated with physical activity, individuals with SCI/D can be empowered to manage their health and prevent modifiable risk factors associated with CMD.
