# Supplementary material for: Developing and Evaluating Data Infrastructure and Implementation Tools to Support Cardiometabolic Disease Indicator Data Collection
Source: Top Spinal Cord Inj Rehabil. 2023 Nov 17;29(Suppl):124–41. doi: 10.46292/sci23-00018S (PMC10759866; doi:10.46292/sci23-00018S)
Supplement: Supplementary file 1 [file i1945-5763-29-suppl-124-s02.pdf]

SCI-HIGH

CARDIOMETABOLIC HEALTH - FINAL OUTCOME INDICATOR (Outpatient)

Using this survey, we intend to document if people living with a spinal cord injury in the community exercise and if these exercises are at appropriate intensity and duration. We are also interested in documenting lipid profiles (e.g. blood cholesterol levels) and prescribed treatments (e.g. drugs, diet, physical exercise). This information will be recorded using the following multiple-choice survey and used to inform the care and education provided during and after spinal cord injury rehabilitation.
The personal information record in this survey will only be seen by Dr. Cathy Craven (project leader) and the SCI-High team. Other project members may see a summary of the information without your personal information. Data will be reported in aggregate form, and you will not be named in any reports or publications now or in the future. The project leader will keep your answers anonymous (without your name or other information that can identify you) over the next three years. All digital information will be stored in secure and encrypted hospital servers.

PARTICIPANT'S INFORMATION

Reachlite ID: CHFO2022DTQUS____

Consortium ID: ____________________

Please, use a dark pen to mark the squares with "Xs" (x) *or* checkmarks (v).

1. What is the first letter of your first name?

A☐ B☐ C☐ D☐ E☐ F☐ G☐ H☐ I☐ J☐ K☐ L☐ M☐

N☐ O☐ P☐ Q☐ R☐ S☐ T☐ U☐ V☐ W☐ X☐ Y☐ Z☐

2. What is the first letter of your last name?

A☐ B☐ C☐ D☐ E☐ F☐ G☐ H☐ I☐ J☐ K☐ L☐ M☐

N☐ O☐ P☐ Q☐ R☐ S☐ T☐ U☐ V☐ W☐ X☐ Y☐ Z☐

3. Do you self-identify as a:

☐ Female ☐ Male ☐ Another gender identity ☐ Prefer not to say

4. What are the first three digits of your current postal code?

First digit	*Second digit*	*Third digit*
A☐ B☐ C☐ D☐ E☐ F☐ G☐	1☐ 2☐ 3☐	A☐ B☐ C☐ D☐ E☐ F☐ G☐
H☐ I☐ J☐ K☐ L☐ M☐ N☐	4☐ 5☐ 6☐	H☐ I☐ J☐ K☐ L☐ M☐ N☐
O☐ P☐ Q☐ R☐ S☐ T☐ U☐	7☐ 8☐ 9☐	O☐ P☐ Q☐ R☐ S☐ T☐ U☐
V☐ W☐ X☐ Y☐ Z☐	0☐	V☐ W☐ X☐ Y☐ Z☐

5. What year were you born?

☐ 1930	☐ 1940	☐ 1950	☐ 1960	☐ 1970	☐ 1980	☐ 1990	☐ 2000
☐ 1931	☐ 1941	☐ 1951	☐ 1961	☐ 1971	☐ 1981	☐ 1991	☐ 2001
☐ 1932	☐ 1942	☐ 1952	☐ 1962	☐ 1972	☐ 1982	☐ 1992	☐ 2002
☐ 1933	☐ 1943	☐ 1953	☐ 1963	☐ 1973	☐ 1983	☐ 1993	☐ 2003
☐ 1934	☐ 1944	☐ 1954	☐ 1964	☐ 1974	☐ 1984	☐ 1994	☐ 2004
☐ 1935	☐ 1945	☐ 1955	☐ 1965	☐ 1975	☐ 1985	☐ 1995	☐ 2005
☐ 1936	☐ 1946	☐ 1956	☐ 1966	☐ 1976	☐ 1986	☐ 1996	☐ 2006
☐ 1937	☐ 1947	☐ 1957	☐ 1967	☐ 1977	☐ 1987	☐ 1997	☐ 2007
☐ 1938	☐ 1948	☐ 1958	☐ 1968	☐ 1978	☐ 1988	☐ 1998	☐ 2008
☐ 1939	☐ 1949	☐ 1959	☐ 1969	☐ 1979	☐ 1989	☐ 1999	☐ 2009

6. How long ago did you have your spinal cord injury/disease?

☐ 0-6 months	☐ 2-5 years	☐ 20-25 years	☐ 35-40 years
☐ 6-12 months	☐ 6-10 years	☐ 25-30 years	☐ >40 years
☐ 12-18 months	☐ 10-15 years	☐ 30-35 years	☐ Don’t know
☐ 18-24 months	☐ 15-20 years		

7. What was the cause of your spinal cord injury/disease? (*pick one*)

☐ Trauma (fall, car accident, etc.) ☐ Nontrauma (disease or surgery)

8. Do you have: (*pick one*) ☐ Paraplegia ☐ Quadriplegia

9. Do you have motor function below the level of injury? ☐ Yes ☐ No

10. Do you have pinprick or light touch sensation below the level of injury? ☐ Yes ☐ No

11. Most of the time, what mobility device do you use outside your home?

☐ Manual wheelchair	☐ Walker	☐ Walking without assistance
☐ Power wheelchair	☐ Cane	☐ Other

QI ID 20-0007 - Oct 27, 2020 ©SCI-High Project/UHN

12. Most of the time, what mobility device do you use inside your home?

☐ Manual wheelchair ☐ Walker ☐ Walking without assistance

☐ Power wheelchair ☐ Cane ☐ Other

AEROBIC PHYSICAL EXERCISE

For this questionnaire, exercise refers to "aerobic exercise" to maintain your heart health. Moderate to vigorous exercise refers to ratings of perceived exertion between 14 (hard) to 17 (very hard - feeling very tired, and pushing to keep going). Some examples of aerobic exercise may include wheelchair pushing, arm cycling, swimming, rowing, and participation in sports.

13. Do you do any moderate to vigorous-intensity exercise (aerobic) to maintain your heart health?

☐ No ☐ Yes ☐ Don't know

If you answered "No", go to question 16.

14. In a regular week, how many days do you do moderate to vigorous-intensity aerobic physical activity for your heart health?

☐ 0 days ☐ 1 days ☐ 2 days ☐ 3 days ☐ 4 days

☐ 5 day ☐ 6 days ☐ 7 days ☐ Don't know

15. In these days that you exercise, how many minutes per day do you spend doing moderate to vigorous-intensity exercise (aerobic) for your heart health?

☐ 0 min/day ☐ 21-40 min/day ☐ More than 60 min/day

☐ 1-20 min/day ☐ 41-60 min/day ☐ Don't know

LIPID PROFILE ASSESSMENT (Cholesterol check)

16. Did you receive a lipid profile assessment (i.e. measure of your blood cholesterol levels and triglycerides) since your discharge from rehabilitation?

☐ No ☐ Yes ☐ Don't know

If you answered "No", you may stop the questionnaire.

17. Did your health care provider (e.g. physiatrist, family doctor, etc.) tell you about your lipid levels (e.g. cholesterol and triglycerides from your blood results)?

☐ Yes, they were normal ☐ Yes, they were abnormal (e.g. too high or too low)

☐ No, they did not tell me ☐ Don't know

If you answered "Yes, they were abnormal (e.g. too high or too low)", please answer question 18, below:

QI ID 20-0007 - Oct 27, 2020 ©SCI-High Project/UHN

18. Did your health care provider (e.g. physiatrist, family doctor, etc.) recommend changes in your care or lifestyle to treat your abnormal lipid levels?
Choose one option.

☐ Yes, physical exercise
☐ Yes, diet
☐ Yes, drugs
☐ Yes, physical exercise and diet
☐ Yes, physical exercise and drugs
☐ Yes, physical exercise, diet, and drugs
☐ Yes, other
☐ No
☐ Don't know

Report Date: Y Y Y Y / M M / D D *For Office use only.*

TEST	Test Completed? (Y/N)	VALUE	LOWER LIMIT REF. RANGE	UPPER LIMIT REF. RANGE	ABNORMAL VALUE (Y/N?)	ACTION REQUIRED
Triglycerides (mmol/L)	□ **Yes**<br>□ **No**				□ **Yes**<br>□ **No**	□ **Yes**<br>□ **No**
Total Cholesterol (mmol/L)	□ **Yes**<br>□ **No**				□ **Yes**<br>□ **No**	□ **Yes**<br>□ **No**
HDL-c (mmol/L)	□ **Yes**<br>□ **No**				□ **Yes**<br>□ **No**	□ **Yes**<br>□ **No**
LDL-c (mmol/L)	□ **Yes**<br>□ **No**				□ **Yes**<br>□ **No**	□ **Yes**<br>□ **No**
Non-HDL-c (mmol/L)	□ **Yes**<br>□ **No**				□ **Yes**<br>□ **No**	□ **Yes**<br>□ **No**
TC/HDL-c	□ **Yes**<br>□ **No**				□ **Yes**<br>□ **No**	□ **Yes**<br>□ **No**

QI ID 20-0007 - Oct 27, 2020 ©SCI-High Project/UHN
