# Supplementary material for: Developing and Evaluating Data Infrastructure and Implementation Tools to Support Cardiometabolic Disease Indicator Data Collection
Source: Top Spinal Cord Inj Rehabil. 2023 Nov 17;29(Suppl):124–41. doi: 10.46292/sci23-00018S (PMC10759866; doi:10.46292/sci23-00018S)
Supplement: Supplementary file 2 [file i1945-5763-29-suppl-124-s03.pdf]

Cardiometabolic Toolkit for People Living with SCI/D

What is cardiometabolic disease (CMD) and what causes it?

- Individuals living with SCI are at increased risk of developing CMD.[2]
- CMD risk has five component risks: obesity, diabetes or pre-diabetes, high blood pressure, low HDL (good) cholesterol, and elevated fats in your blood.[1]
- CMD is caused or worsened by a mismatch between what and how much you eat, and not enough physical activity.[1] In other words, if you consume more calories (especially in saturated fats) than you burn, then you are at more risk for CMD.[1]
- Physical activity and maintaining an optimal body weight can mitigate CMD risk.[1]

Table 1. Guideline Definition of CMD [1,a]

	**Diagnosis**	**Value**
☐	Body Mass Index	≥22 kg/m^2; or greater than 22% body fat on whole-body DXA scan
☐	Plasma Triglycerides (fats)	≥ 150 mg/dL (1.7 mmol/L)
☐	Reduced HDL (“good”) cholesterol	Men: < 40 mg/dL (1.03 mmol/L)<br>Women: < 50 mg/dL (1.29 mmol/L)
☐	Elevated blood pressure	≥ 130/85 mmHg or use of medication for hypertension
☐	Fasting glucose	≥ 100 mg/dL (5.6 mmol/L) or use of medication for hyperglycemia
☐	*Elevated C-reactive protein (CRP)	Lifelabs: ≥2 mg/L<br>Dynacare: >3.0 mg/L<br>UHN: ≥2 mg/L<br>Alpha Labs: >3.0 mg/L
☐	*Waist Circumference	Men: > 40 inches (102 cm)<br>Women: > 35 inches (88 cm)

*additional component risks to the definition for CMD

- Greater than or equal to 3 from this list of criteria is defined as CMD.[1]
- In addition, non-traditional component risks of cardiometabolic disease include family history, genetics, and the tendency to develop blood clots in your veins or develop fatty plaques in your arteries.

Why is it important for you to reduce the risk of CMD:

- CMD risk promotes heart disease and Type 2 diabetes mellitus. These diseases narrow or block blood vessels that can result in chest pain, heart attacks, or stroke.[1]
- Heart disease is one of the leading causes of death in the chronic SCI population, contributing to half of all deaths.[2]

Interventions for CMD:

1) Staying Active

Note - please talk to your doctor, physiotherapist, or exercise specialist to see if exercise is safe for you and to get any specific instructions

- Whether you are a new exerciser or advanced exerciser, everyone needs to do a warm-up before exercising their heart and cool-down after exercise.

New Exercisers - those who have not participated in exercise previously or those who have been inactive for extended periods.[3]

Advanced Exercisers - those who are currently active and accustomed to aerobic and resistance-based activities.[3]

- **Warm-up:** A sufficient warm-up of 5-10 minutes should be incorporated before performing any exercise. Warm up should be progressive, from slow small movements to larger and more intense. It should be inclusive of as many active muscles as possible in continuous fashion (repetitive and rhythmic) to keep body dynamic and stimulate muscles, heart and lungs.[3]

- **New Exercisers**: 20 minutes of moderate to vigorous intensity aerobic exercise 2x/week AND 3 sets of strength exercises for each major functioning muscle group, at a moderate to vigorous intensity, 2x/ week are necessary to improve cardiorespiratory fitness and muscle strength.[2,3]

- **Advanced Exercisers**: At least 30 minutes of moderate to vigorous intensity aerobic exercise three times per week for cardiometabolic health benefits.[2,3]

- **Cool-down:** Cool down should be incorporated at the end of the exercise protocol. It should be progressive (opposite to warm up), also including as many active muscles as possible in continuous fashion with both dynamic and static stretching.[3]

Types of Exercise

- **Aerobic exercise** - Physical activities that are done continuously and that increase your heart rate and breathing rate, such as wheeling, swimming, cycling or dancing.[4]
- **Resistance exercise** - Strength-training activities that increase muscle strength, such as exercises using resistance bands, resistance machines, or lifting weights.[4]

Intensity of Exercise

- **Moderate Intensity –** Physical activities that require you to work somewhat hard, but you should feel like you can keep going for a long time. You should be able to talk comfortably during these activities, but not sing.[4]
- **Vigorous Intensity** – Physical activities that require you to work really hard, and you can only continue them for a short time before getting tired.[4]

2) Dietary Interventions

Please consult your healthcare provider or dietician for specific meal plans

- All individuals should adopt a heart-healthy nutrition plan focusing on fruits, vegetables, whole grains, low-fat dairy, poultry, fish, legumes, non-tropical vegetable oils, and nuts, while limiting sweets, sugar-sweetened beverages, and red meats.[1]
- The Dietary Approach to Stop Hypertension (DASH) nutritional plan or Mediterranean diet should be adopted if high blood pressure or additional cardiometabolic risk factors are present.[1] (Please see handout "Eating Healthy")
- Saturated fat should be limited to 5-6% of your total caloric intake.[1]
- Daily sodium intake should be limited to ≤ 2400 mg for individuals with high blood pressure

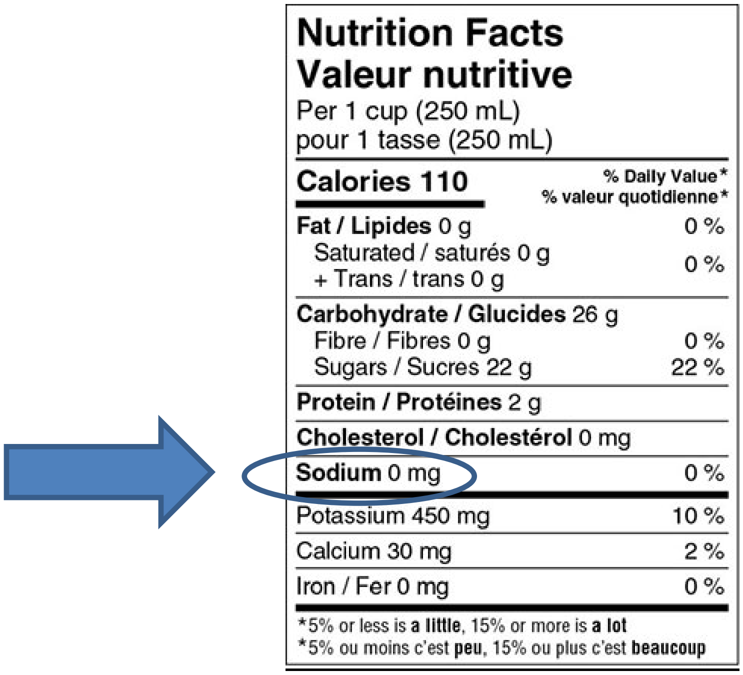

TALK TO YOUR FAMILY DOCTOR ABOUT YOUR LIPID PROFILE (BLOOD CHOLESTEROL LEVELS) AND DIABETES MANAGEMENT TO LOWER YOUR RISK OF CMD.

References

1. Paralyzed Veterans of America. Identification and Management of Cardiometabolic Risk after Spinal Cord Injury Clinical Practice Guideline for Health Care Providers. Paralyzed Veterans of America. Accessed October 25th, 2021. https://pva.org/wp-content/uploads/2021/09/cpg_cardiometabolic-risk_digital.pdf

2. Wiest MJ, West C, Ditor D, Furlan JC, Miyatani M, Farahani F, S Mohammad A, Oh PI, Bayley MT, Craven BC. Development of Cardiometabolic Health indicators to advance the quality of spinal cord injury rehabilitation: SCI-High Project, The Journal of Spinal Cord Medicine, 2019;42(1): 166-175. doi:10.1080/10790268.2019.1613322

3. Rowan C. EXERCISE PRESCRIPTION FOR SPINAL CORD INJURY (SCI). KITE-UHN. Accessed October 27, 2021. https://kite-uhn.com/e-learning/en/exercise-prescription-for-spinal-cord-injury

4. The University of British Columbia Okanagan Campus. Physical Activity Guidelines for Adults with Spinal Cord Injury. The University of British Columbia SCI Guidelines. Updated May 29, 2019. Accessed November 10, 2021. https://sciguidelines.ubc.ca/
