# Supplementary material for: Developing and Evaluating Data Infrastructure and Implementation Tools to Support Cardiometabolic Disease Indicator Data Collection
Source: Top Spinal Cord Inj Rehabil. 2023 Nov 17;29(Suppl):124–41. doi: 10.46292/sci23-00018S (PMC10759866; doi:10.46292/sci23-00018S)
Supplement: Supplementary file 3 [file i1945-5763-29-suppl-124-s04.pdf]

Lyndhurst Centre
520 Sutherland Drive
Toronto, ON
M4G 3V9

Insert patient label here

Quality Improvement (QI): Cardiometabolic Health Patient Interview

Resources Given**:**

- ☐ SCI Action Canada Exercise Guidelines
- ☐ CMD Patient Handout
- ☐ 100km Tune-up Checklist
- ☐ List of Accessible and Inclusive Fitness Centres
- ☐ Medication Handouts
- ☐ Healthy Eating on a Budget
- ☐ Other:____________________

Notes/Recommendations for Patient:

Follow-up/Action Items

________________________________ ________________________________
Signature of Staff Member Date: YYYY/MM/DD
