# Supplementary material for: Developing and Evaluating Data Infrastructure and Implementation Tools to Support Cardiometabolic Disease Indicator Data Collection
Source: Top Spinal Cord Inj Rehabil. 2023 Nov 17;29(Suppl):124–41. doi: 10.46292/sci23-00018S (PMC10759866; doi:10.46292/sci23-00018S)
Supplement: Supplementary file 4 [file i1945-5763-29-suppl-124-s05.pdf]

LIPID PROFILE CHECKLIST (PRIMARY VS SECONDARY PREVENTION) – OFFICE USE ONLY

Consortium ID

☐Primary
☐Secondary

Use this checklist to determine if primary or secondary lipid targets are appropriate.

Check to see if the patient has ANY of the following?

If YES, to any criteria, check the box and use the secondary prevention lipid targets.
*Once you have identified one risk factor you can **stop** completing the checklist***.**
If NO to all, use the primary prevention targets

Family History:

☐ Family history of heart attack/stroke before age 55 for men/ age 65 for women
☐Family history of dyslipidemia

Current History:

☐Obesity (BMI ≥ 30)
☐ Diabetes Mellitus
☐ Hypertension BP > 140/90mmHg on three occasions in three different settings
☐ Current cigarette smoking
☐Chronic Kidney Disease (CKD -eGFR ≤ 60 mL/min/1.73 m^2 or ACR ≥ 3 mg/mmol)
☐History of stable or unstable angina, MI, TIA, stroke, positive angiogram, revascularization procedure, carotid stenosis, or peripheral artery disease, abdominal aortic aneurysm
☐ Any of the following conditions:
    Rheumatoid Arthritis
    Lupus
    Psoriatic Arthritis
    Ankylosing Spondylitis
    Inflammatory Bowel Disease
☐Erectile Dysfunction prior to SCI onset
☐COPD
☐HIV Infection
☐History of hypertension during pregnancy

If in Person:

☐Stigmata of dyslipidemia (corneal arcus, xanthelasma, xanthoma)
https://dermnetnz.org/topics/xanthoma

References

1. Pearson, G.J., et al., *2021 Canadian Cardiovascular Society Guidelines for the Management of Dyslipidemia for the Prevention of Cardiovascular Disease in Adults.* Can J Cardiol, 2021. 37(8): p. 1129-1150.

Updated – April 22nd, 2022
