# Supplementary material for: Developing and Evaluating Data Infrastructure and Implementation Tools to Support Cardiometabolic Disease Indicator Data Collection
Source: Top Spinal Cord Inj Rehabil. 2023 Nov 17;29(Suppl):124–41. doi: 10.46292/sci23-00018S (PMC10759866; doi:10.46292/sci23-00018S)
Supplement: Supplementary file 5 [file i1945-5763-29-suppl-124-s06.pdf]

Toronto Rehabilitation Institute
The Kite Research Institute

SCI-HIGH PROJECT
SPINAL CORD INJURY (SCI) REHABILITATION CARE HIGH PERFORMANCE INDICATORS

LIPID PROFILE RANGES (PRIMARY VS SECONDARY PREVENTION) – OFFICE USE ONLY

**Lifelabs**	**High or Intermediate CVD risk (Secondary Prevention)**	
	Reference Range	Triglycerides: < 1.7 mmol/L
		Total Cholesterol: ≤ 5.2mmol/L
		HDL-C: Men: ≥ 1.03 mmol/L<br>Women: ≥ 1.29 mmol/L
		LDL-C: < 1.8 mmol/L<br>(Use non-HDL if fasting ≤ 10 hours or Triglycerides ≥ 1.5 mmol/L)
		Non-HDL: < 2.4mmol/L
		TC/HDL-C: ≤ 4.5mmol/L
	**Low CVD risk (Primary Prevention)**	
	Reference Range	Triglycerides: < 1.7 mmol/L
		Total Cholesterol: ≤ 5.2mmol/L
		HDL-C: Men: ≥ 1.03 mmol/L<br>Women: ≥ 1.29 mmol/L
		LDL-C: ≤ 2.0 mmol/L<br>(Use non-HDL if fasting ≤ 10 hours or Triglycerides ≥ 1.5 mmol/L)
		Non-HDL: ≤ 2.6mmol/L
		TC/HDL-C: ≤ 4.5mmol/L

**Dynacare**	**High or Intermediate CVD risk (Secondary Prevention)**	
	Reference Range	Triglycerides: < 1.7 mmol/L
		Total Cholesterol: ≤ 5.2 mmol/L
		HDL-C: Men: ≥ 1.0 mmol/L<br>Women: ≥ 1.30 mmol/L
		LDL-C: < 1.8 mmol/L<br>(Use non-HDL if fasting ≤ 10 hours or Triglycerides ≥ 1.5 mmol/L)
		Non-HDL < 2.4mmol/L
		TC/HDL-C: ≤ 4.5mmol/L
	**Low CVD risk (Primary Prevention)**	
	Reference Range	Triglycerides: < 1.7 mmol/L
		Total Cholesterol: ≤ 5.2 mmol/L
		HDL-C: Men: ≥ 1.0 mmol/L<br>Women: ≥ 1.30 mmol/L
		LDL-C: < 2.0 mmol/L<br>((Use non-HDL if fasting ≤ 10 hours or Triglycerides ≥ 1.5 mmol/L)
		Non-HDL ≤ 2.6mmol/L
		TC/HDL-C: ≤ 4.5mmol/L

LIPID PROFILE RANGES (PRIMARY VS SECONDARY PREVENTION) – OFFICE USE ONLY

UHN	**High or Intermediate CVD risk (Secondary Prevention)**	
	Reference Range	Triglycerides: < 1.7 mmol/L
		Total Cholesterol: ≤ 5.2mmol/L
		HDL-C: Men: ≥ 1.03 mmol/L<br>Women: ≥ 1.29 mmol/L
		LDL-C: < 1.8 mmol/L<br>(Use non-HDL if fasting ≤ 10 hours or Triglycerides ≥ 1.5 mmol/L)
		Non-HDL: < 2.4mmol /L
		TC/HDL-C: ≤ 4.5mmol/L
	**Low CVD risk (Primary Prevention)**	
	Reference Range	Triglycerides: < 1.7 mmol/L
		Total Cholesterol: ≤ 5.2mmol/L
		HDL-C: Men: ≥ 1.03 mmol/L<br>Women: ≥ 1.29 mmol/L
		LDL-C: < 2.0 mmol/L<br>(Use non-HDL if fasting ≤ 10 hours or Triglycerides ≥ 1.5 mmol/L)
		Non-HDL: ≤ 2.6mmol/L
		TC/HDL-C: ≤ 4.5mmol/L

References

1. Pearson, G.J., et al., *2021 Canadian Cardiovascular Society Guidelines for the Management of Dyslipidemia for the Prevention of Cardiovascular Disease in Adults.* Can J Cardiol, 2021. 37(8): p. 1129-1150.

2. Lifelabs. *Lipid Assessment*. 2022 April 27, 2022]; Available from: https://tests.lifelabs.com/.

3. Dynacare. *TOTAL CHOLESTEROL/HDL RATIO (Ontario).* 2022 April 27, 2022]; Available from: https://www.dynacare.ca/.

4. Koyuncu, E., et al., *The analysis of serum lipid levels in patients with spinal cord injury.* J Spinal Cord Med, 2017. **40**(5): p. 567-572.
