# Supplementary material for: Developing and Evaluating Data Infrastructure and Implementation Tools to Support Cardiometabolic Disease Indicator Data Collection
Source: Top Spinal Cord Inj Rehabil. 2023 Nov 17;29(Suppl):124–41. doi: 10.46292/sci23-00018S (PMC10759866; doi:10.46292/sci23-00018S)
Supplement: Supplementary file 6 [file i1945-5763-29-suppl-124-s07.pdf]

UHN Toronto Rehabilitation Institute
The Kite Research Institute

SCI-HIGH PROJECT
SPINAL CORD INJURY (SCI) REHABILITATION CARE HIGH PERFORMANCE INDICATORS

LIPID PROFILE ABNORMALITIES (PRIMARY VS SECONDARY PREVENTION) – OFFICE USE ONLY

**Lifelabs**	**High or Intermediate CVD risk (Secondary Prevention)**	
	Abnormalities	Triglycerides: ≥ 1.7 mmol/L
		Total Cholesterol: > 5.2 mmol/L
		HDL-C: Men: < 1.03 mmol/L<br>Women: < 1.29 mmol/L
		LDL-C: ≥ 1.8 mmol/L<br>(Use non-HDL if fasting ≤ 10 hours or Triglycerides ≥ 1.5 mmol/L)
		Non-HDL ≥ 2.4 mmol/L
		TC/HDL-C: > 4.5
	**Low CVD risk (Primary Prevention)**	
	Abnormalities	Triglycerides: ≥ 1.7 mmol/L
		Total Cholesterol: > 5.2 mmol/L
		HDL-C: Men: < 1.03 mmol/L<br>Women: < 1.29 mmol/L
		LDL-C: ≥ 3.5 mmol/L<br>(Use non-HDL if fasting ≤ 10 hours or Triglycerides ≥ 1.5 mmol/L)
		Non-HDL ≥ 4.2 mmol/L
		TC/HDL-C: > 4.5

**Dynacare**	**High or Intermediate CVD risk (Secondary Prevention)**	
	Abnormalities	Triglycerides: ≥ 1.7 mmol/L
		Total Cholesterol: > 5.2 mmol/L
		HDL-C: Men: < 1.03 mmol/L<br>Women: < 1.29 mmol/L
		LDL-C ≥ 1.8 mmol/L<br>(Use non-HDL if fasting ≤ 10 hours or Triglycerides ≥ 1.5 mmol/L)
		Non-HDL ≥ 2.4 mmol/L
		TC/HDL-C: > 4.5
	**Low CVD risk (Primary Prevention)**	
	Abnormalities	Triglycerides ≥ 1.7 mmol/L
		Total Cholesterol > 5.2 mmol/L
		HDL-C: Men: < 1.03 mmol/L<br>Women: < 1.29 mmol/L
		LDL-C: ≥ 3.5 mmol/L<br>(Use non-HDL if fasting ≤ 10 hours or Triglycerides ≥ 1.5 mmol/L)
		Non-HDL ≥ 4.2 mmol/L
		TC/HDL-C: ≥ 5.0

LIPID PROFILE ABNORMALITIES (PRIMARY VS SECONDARY PREVENTION) – OFFICE USE ONLY

**UHN**	**High or Intermediate CVD risk (Secondary Prevention)**	
	Abnormalities	Triglycerides: ≥ 1.7 mmol/L
		Total Cholesterol: > 5.2 mmol/L
		HDL-C: Men: < 1.03 mmol/L<br>Women: < 1.29 mmol/L
		LDL-C: ≥ 1.8 mmol/L<br>(Use non-HDL if fasting ≤ 10 hours or Triglycerides ≥ 1.5 mmol/L)
		Non-HDL ≥ 2.4 mmol/L
		TC/HDL-C: > 4.5
	**Low CVD risk (Primary Prevention)**	
	Abnormalities	Triglycerides: ≥ 1.7 mmol/L
		Total Cholesterol: > 5.2 mmol/L
		HDL-C: Men: < 1.03 mmol/L<br>Women: < 1.29 mmol/L
		LDL-C: ≥ 3.5 mmol/L<br>(Use non-HDL if fasting ≤ 10 hours or Triglycerides ≥ 1.5 mmol/L)
		Non-HDL ≥ 4.3 mmol/L
		TC/HDL-C: > 4.5

References

1. Pearson, G.J., et al., *2021 Canadian Cardiovascular Society Guidelines for the Management of Dyslipidemia for the Prevention of Cardiovascular Disease in Adults.* Can J Cardiol, 2021. 37(8): p. 1129-1150.

2. Lifelabs. *Lipid Assessment*. 2022 April 27, 2022]; Available from: https://tests.lifelabs.com/.

3. Dynacare. *TOTAL CHOLESTEROL/HDL RATIO (Ontario).* 2022 April 27, 2022]; Available from: https://www.dynacare.ca/.

4. Koyuncu, E., et al., *The analysis of serum lipid levels in patients with spinal cord injury.* J Spinal Cord Med, 2017. **40**(5): p. 567-572.
