# Supplementary material for: Developing and Evaluating Data Infrastructure and Implementation Tools to Support Cardiometabolic Disease Indicator Data Collection
Source: Top Spinal Cord Inj Rehabil. 2023 Nov 17;29(Suppl):124–41. doi: 10.46292/sci23-00018S (PMC10759866; doi:10.46292/sci23-00018S)
Supplement: Supplementary file 7 [file i1945-5763-29-suppl-124-s08.pdf]

Medications for Cardiometabolic Health

ROSUVASTATIN
(CRESTOR 10-20MG)

Indication:

- Rosuvastatin is used along with a good diet to help lower "bad" cholesterol and fats (such as LDL, triglycerides) and raise "good" cholesterol (HDL) in the blood.[1] It belongs to a group of drugs known as statins.[1]
- Rosuvastatin works by reducing the amount of cholesterol made by the liver. Lowering "bad" cholesterol and triglycerides and raising "good" cholesterol decreases the risk of cardiovascular outcomes such as heart attack and stroke.[1]
- Rosuvastatin has been shown to reduce all-cause mortality in individuals with heart disease, or diabetes without heart disease, in the general population.[2]

Directions for Use:

1. Take this medication by mouth with or without food as directed by your doctor, usually once daily at night.[1]
2. Swallow the capsules whole without crushing or chewing. If you have trouble swallowing talk to your pharmacist about how to take the medication.[1]
3. If you miss a dose, take your next dose at the usual time and <u>do not double up to make up for the missed dose.</u>[1]
4. Keep this medication in the container it came in, tightly closed, and out of reach of children. Store it at room temperature and away from excess heat and moisture (not in the bathroom).[1]

Potential Side Effects:

- Less severe side effects include: constipation, decreased sexual ability, difficulty sleeping, dizziness, headache, nausea, nightmares, and stomach pain. Please contact your doctor if these symptoms don't go away.[3]
- Get medical help immediately if you experience any of the following symptom[3]:

  1) Breast enlargement
  2) Confusion
  3) Itching, numbness, tingling, weakness, or pain in the hands or feet
  4) Persistent cough, with or without shortness of breath
  5) Poor memory
  6) Signs of clotting problems (e.g., unusual nosebleeds, bruising, blood in urine, coughing blood, bleeding gums, cuts that don't stop bleeding)
  7) Signs of depression (e.g., poor concentration, changes in weight, changes in sleep, decreased interest in activities, thoughts of suicide)
  8) Symptoms of high blood sugar (e.g., frequent urination, increased thirst, excessive eating, unexplained weight loss, poor wound healing, infections, fruity breath odour)
  9) Symptoms of liver damage (such as yellow skin or eyes, abdominal pain, dark urine, clay-coloured stools, loss of appetite, nausea and vomiting, or itching)
  10) Symptoms of muscle damage (unexplained muscle pain, tenderness or weakness, or brown or discoloured urine – especially if you also have a fever or a general feeling of being unwell
  11) Difficulty swallowing or breathing
  12) Mild or severe skin rash, including skin blistering and peeling (possibly with headache, fever, coughing, or aching before the rash begins)
  13) Signs of pancreatitis (e.g., abdominal pain on the upper left side, back pain, nausea, fever, chills, rapid heartbeat, swollen abdomen)
  14) Symptoms of a serious allergic reaction (such as swelling of the face or throat, hives, or difficulty brea

ROSUVASTATIN: Precautions/Contraindications Checklist

**Contraindications :**	
	Female patients who are pregnant. Do not breastfeed while taking this medication.
**Precaution & relevant warnings:**	
	Allergy to any of the ingredients?
	Taking aluminum and magnesium hydroxide antacids (Mylanta, Maalox)? Take them at least 2 hours after rosuvastatin.
	Taking any of the following medications: apalutamide, bezafibrate, carbamazepine, clopidogrel, cobicistat, colchicine, cyclosporine, dronedarone, elagolix, eltrombopag, eslicarbazepine, fenofibrate, gemfibrozil, hepatitis C antivirals (e.g., glecaprevir and pibrentasvir, grazoprevir, ledipasvir, sofosbuvir, velpatasvir, voxilaprevir), HIV protease inhibitors (e.g., atazanavir, indinavir, ritonavir, saquinavir), itraconazole, letermovir, niacin (nicotinic acid), niacinamide, pazopanib, raltegravir, regorafenib, repaglinide, rifampin, other "statin" anti-cholesterol medications (e.g., atorvastatin, lovastatin, simvastatin), teriflunomide, tolvaptan, trabectedin, warfarin? Please consult with your doctor about drug interactions.
	Liver disease?
	Family history of muscle disease or muscle toxicity?
	Having surgery or hospitalized due serious injury?
	Consuming alcohol? Can increase risk of serious side effects.

References:

1. Canadian Pharmacists Association. Rosuvastatin. CPS. Updated May 14th, 2020. Accessed December 4th 2021. https://www-e-therapeutics-ca.uhn.idm.oclc.org/search

2. Paralyzed Veterans of America. Identification and Management of Cardiometabolic Risk after Spinal Cord Injury Clinical Practice Guideline for Health Care Providers. Paralyzed Veterans of America. Accessed October 25th, 2021. https://pva.org/wp-content/uploads/2021/09/cpg_cardiometabolic-risk_digital.pdf

3. MediResource. Crestor. MedBroadcast. Accessed December 4th, 2021. https://www.medbroadcast.com/drug/getdrug/crestor
