# Supplementary material for: Developing and Evaluating Data Infrastructure and Implementation Tools to Support Cardiometabolic Disease Indicator Data Collection
Source: Top Spinal Cord Inj Rehabil. 2023 Nov 17;29(Suppl):124–41. doi: 10.46292/sci23-00018S (PMC10759866; doi:10.46292/sci23-00018S)
Supplement: Supplementary file 8 [file i1945-5763-29-suppl-124-s09.pdf]

Medications for Cardiometabolic Health

FENOFIBRATE
(48-200MG)

Indication:

- Fenofibrate is used with a good diet, exercise, and sometimes with other medications to help lower "bad" cholesterol and fats (such as LDL, triglycerides) and raise "good" cholesterol (HDL) in the blood.[1]
- It belongs to a class of medications called antilipemic agents. It works by speeding the natural processes that remove cholesterol from the body.[1]
- Fenofibrate has shown to decrease the risk of cardiovascular outcomes in the general healthy population.[2]

Directions for Use:

1. Fenofibrate comes as a capsule, a delayed-release (long-acting) capsule, and a tablet to take by mouth. It is usually taken once a day.[1]
2. Take this medication with or without food as directed by your doctor or pharmacist.[1]
3. Swallow the tablet or capsule whole without splitting, chewing, or crushing them.[1]
4. If you miss a dose, take your next dose at the usual time and do not double up to make up for the missed dose.[1]
5. Keep this medication in the container it came in, tightly closed, and out of reach of children. Store it at room temperature and away from excess heat and moisture (not in the bathroom).[1]

Potential Side Effects:

- Less severe side effects include: constipation, decreased interest in sexual activity, diarrhea, dizziness, fatigue, gas, hair loss, headache, increased sensitivity of skin to sunlight, joint pain, nausea, red, itchy skin, skin rash, stomach pain, trouble sleeping, vomiting, and weight loss. Please contact your doctor if these symptoms don't go away.[3]
- Get medical help immediately if you experience any of the following symptoms:
  1) Signs of liver problems such as dark urine, general feeling of being unwell, loss of appetite, nausea, vomiting, or yellowing of the eyes or skin.
  2) Signs of muscle damage (e.g., unexplained muscle pain, tenderness or weakness, or brown or discoloured urine).
  3) Signs of a blood clot in the arm or leg (tenderness, pain, swelling, warmth, or redness in the arm or leg) or lungs (difficulty breathing, sharp chest pain that is worse when breathing in, coughing, coughing up blood, sweating, or passing out)
  4) Signs of pancreatitis (e.g., abdominal pain on the upper left side, back pain, nausea, fever, chills, rapid heartbeat, swollen abdomen)
  5) Signs of a serious allergic reaction (e.g., abdominal cramps, difficulty breathing, nausea and vomiting, or swelling of the face and throat)

FENOFIBRATE: Precautions/Contraindications Checklist

**Contraindications :**	
	Female patients who are pregnant. Do not breastfeed while taking this medication.
**Precaution & relevant warnings:**	
	Allergy to any of the ingredients?
	Taking a bile acid resin such as cholestyramine (Questran), colesevelam (WelChol), or colestipol (Colestid)? Take 2 hours before or after taking fenofibrate.
	Taking any of the following medications:colchicine, cyclosporine, erlotinib, everolimus, ezetimibe, raltegravir, rosiglitazone, "statin" medications (e.g., atorvastatin, lovastatin, simvastatin), sulfonylurea diabetes medications (e.g., gliclazide, glyburide), tacrolimus, ursodiol, warfarin? Please consult with your doctor about drug interactions.
	Kidney, liver, heart, pancreatic, or gallbladder disease?
	Having surgery or hospitalized due serious injury?
	Consuming alcohol? Can increase risk of serious side effects.

References:

1. Canadian Pharmacists Association. Lipidil. CPS. Updated February 17th, 2021. Accessed December 4th 2021. https://www.e-therapeutics.ca/search#m143800n00113

2. Paralyzed Veterans of America. Identification and Management of Cardiometabolic Risk after Spinal Cord Injury Clinical Practice Guideline for Health Care Providers. Paralyzed Veterans of America. Accessed October 25th, 2021. https://pva.org/wp-content/uploads/2021/09/cpg_cardiometabolic-risk_digital.pdf

3. MediResource. Crestor. MedBroadcast. Accessed December 4th, 2021. https://www.medbroadcast.com/drug/getdrug/sandoz-fenofibrate-e
