# Supplementary material for: Developing and Evaluating Data Infrastructure and Implementation Tools to Support Cardiometabolic Disease Indicator Data Collection
Source: Top Spinal Cord Inj Rehabil. 2023 Nov 17;29(Suppl):124–41. doi: 10.46292/sci23-00018S (PMC10759866; doi:10.46292/sci23-00018S)
Supplement: Supplementary file 9 [file i1945-5763-29-suppl-124-s10.pdf]

Medications for Cardiometabolic Health

Niaspan
(1500-2000mg)

Indication:

- Niaspan is used along with a good diet to help lower "bad" cholesterol and fats (such as LDL, triglycerides) and raise "good" cholesterol (HDL) in the blood.[1]
- This medication has shown to be more effective and practical in improving cholesterol levels in patients with quadriplegia when compared to exercise and diet alone.[1]

Directions for Use:

1. Take this medication by mouth once a day at night, after a low fat snack.[1]
2. Swallow the tablet whole without splitting, chewing, or crushing them.[1]
3. If you miss a dose, take your next dose at the usual time and do not double up to make up for the missed dose.[1]
4. Keep this medication in the container it came in, tightly closed, and out of reach of children. Store it at room temperature and away from excess heat and moisture (not in the bathroom).[1]

Potential Side Effects:

- Less severe side effects include: abdominal pain, diarrhea, dizziness, drowsiness, dry mouth, flu-like symptoms (e.g., fever, chills, sweating, muscle aches, tiredness),fluid retention (swelling of the hands, feet, or ankles), flushing (warmth, redness, itching, or tingling) of the face, neck, chest, and back, headache, heartburn, increased cough, nausea, rapid or pounding heartbeat, rash, ringing in the ears, sweating, tiredness, vomiting, weakness. Please contact your doctor if these symptoms don't go away.[2]
- Get medical help immediately if you experience any of the following symptoms[2]:
  1) Fainting
  2) Joint pain or muscle pains/weakness
  3) Severe headache (migraine)
  4) Signs of clotting problems (e.g., unusual nosebleeds, bruising, blood in urine, coughing blood, bleeding gums, cuts that don't stop bleeding)
  5) Signs of liver problems (e.g., yellow eyes or skin, abdominal pain, dark urine or pale stools, nausea, vomiting)
  6) Chest pain
  7) Signs of a serious allergic reaction (swelling of the face, tongue, or throat; hives; difficulty breathing)
  8) Symptoms of bleeding in the stomach or intestines (dark, tarry stools; blood coming from rectum; vomiting blood or material that looks like coffee grounds; fast heartbeat; weakness or fainting)

NIASPAN: Precautions/Contraindications Checklist

**Contraindications :**	
	Patients with severe hypotension (low blood pressure).
**Precaution & relevant warnings:**	
	Allergy to any of the ingredients?
	Taking any of the following medications: acetylsalicylic acid (ASA), bile acid sequestrants (drugs that prevent cholesterol reabsorption by the body; cholestyramine, colestipol), certain blood pressure medications (e.g., metoprolol, propranolol, verapamil, diltiazem), diabetes medications (e.g., chlorpropamide, glipizide, glyburide, insulin, metformin, nateglinide, rosiglitazone), nitrates (e.g., nitroglycerin), "statin" cholesterol medications (e.g., atorvastatin, lovastatin, simvastatin), vitamins or nutritional supplements containing niacin, nicotinic acid, or nicotinamide, warfarin? Please consult your doctor about drug interactions.
	Have or had diabetes, kidney, liver, peptic ulcer disease, or gallbladder disease?
	Have or had jaundice (yellowing of the skin or eyes), gout, ulcers, or bleeding problems?
	Having surgery or hospitalized due serious injury?
	Consuming alcohol? Can increase risk of serious side effects.

References:

1. Nash, Mark S., John E. Lewis, Trevor A. Dyson-Hudson, Yaga Szlachcic, Florence Yee, Armando J. Mendez, Ann M. Spungen, and William A. Bauman. Safety, Tolerance, and Efficacy of Extended-Release Niacin Monotherapy for Treating Dyslipidemia Risks in Persons With Chronic Tetraplegia: A Randomized Multicenter Controlled Trial. Archives of Physical Medicine and Rehabilitation, 2011;92(3): 399–410. doi:10.1016/J.APMR.2010.06.029.

2. MediResource. Niaspan. MedBroadcast. Accessed December 4th, 2021. https://www.medbroadcast.com/drug/getdrug/niaspan
