# Supplementary material for: Developing and Evaluating Data Infrastructure and Implementation Tools to Support Cardiometabolic Disease Indicator Data Collection
Source: Top Spinal Cord Inj Rehabil. 2023 Nov 17;29(Suppl):124–41. doi: 10.46292/sci23-00018S (PMC10759866; doi:10.46292/sci23-00018S)
Supplement: Supplementary file 10 [file i1945-5763-29-suppl-124-s11.pdf]

Medications for Cardiometabolic Health

COENZYME Q-10
(100-300mg)

Indication:

- Elevated CRP or prevention of myopathy for patients given statins.[1,2]

Potential Side Effects:

- Palpitations
- Nausea, Vomiting, or diarrhea
- Uncomplicated Urinary Infection
- Headache or insomnia
- Worsening depression

COENZYME Q-10: Precautions Checklist

**Precaution & relevant warnings:**	
	Taking antihypertensive drugs? May lower blood pressure further.
	Taking Warfarin? May decrease the effects of Warfarin

References:

1. Therapeutic Research Centre. Coenzyme Q-10. Natural Medicines. Updated May 14th, 2020. Accessed January 11th, 2022. https://naturalmedicines-therapeuticresearch-com.uhn.idm.oclc.org/databases/food,-herbs-supplements/professional.aspx?productid=938à

2. Garrido-Maraver J, Cordero MD, Oropesa-Avila M, Vega AF, de la Mata M, Pavon AD, Alcocer-Gomez E, Calero CP, Paz MV, Alanis M, de Lavera I, Cotan D, Sanchez-Alcazar JA. Clinical applications of coenzyme Q10. Front Biosci (Landmark Ed). 2014 Jan 1;19:619-33. doi: 10.2741/4231. PMID: 24389208.
